# Recognition Profile of Emotions in Natural and Virtual Faces

**DOI:** 10.1371/journal.pone.0003628

**Published:** 2008-11-05

**Authors:** Miriam Dyck, Maren Winbeck, Susanne Leiberg, Yuhan Chen, Rurben C. Gur, Klaus Mathiak

**Affiliations:** 1 Department of Psychiatry and Psychotherapy, RWTH Aachen University, Aachen, Germany; 2 Center for Social Neuroscience and Neuroeconomics, University of Zürich, Zürich, Switzerland; 3 Department of Psychiatry, University of Pennsylvania, Philadelphia, Pennsylvania, United States of America; 4 Institute of Psychiatry, King's College London, London, United Kingdom; James Cook University, Australia

## Abstract

**Background:**

Computer-generated virtual faces become increasingly realistic including the simulation of emotional expressions. These faces can be used as well-controlled, realistic and dynamic stimuli in emotion research. However, the validity of virtual facial expressions in comparison to natural emotion displays still needs to be shown for the different emotions and different age groups.

**Methodology/Principal Findings:**

Thirty-two healthy volunteers between the age of 20 and 60 rated pictures of natural human faces and faces of virtual characters (avatars) with respect to the expressed emotions: happiness, sadness, anger, fear, disgust, and neutral. Results indicate that virtual emotions were recognized comparable to natural ones. Recognition differences in virtual and natural faces depended on specific emotions: whereas disgust was difficult to convey with the current avatar technology, virtual sadness and fear achieved better recognition results than natural faces. Furthermore, emotion recognition rates decreased for virtual but not natural faces in participants over the age of 40. This specific age effect suggests that media exposure has an influence on emotion recognition.

**Conclusions/Significance:**

Virtual and natural facial displays of emotion may be equally effective. Improved technology (e.g. better modelling of the naso-labial area) may lead to even better results as compared to trained actors. Due to the ease with which virtual human faces can be animated and manipulated, validated artificial emotional expressions will be of major relevance in future research and therapeutic applications.

## Introduction

The recognition of emotions from others' faces is a universal and fundamental skill for social interaction [1], [2]. Increasing research has been dedicated to psychophysics, neural processing and impairments of emotion recognition. In most instances those studies applied still photographs of facial expressions as experimental stimuli. However, these static images may not necessarily reflect the liveliness and true form of dynamic facial expressions as they occur in daily life [3]. Accordingly, recent imaging studies indicate that neural activity is enhanced and more distributed when dynamically morphed relative to static facial expressions are presented to subjects [4], [5]. Likewise, spontaneous facial mimicry is more prominent in response to dynamic relative to static presentations of facial emotions [6].

Virtual reality (VR) has the potential to provide almost realistic, three-dimensional environments created by computer graphics, with which the user can interact. Additionally, it offers a flexible and controlled setting appropriate for application in experimental and therapeutic contexts [7]. Consequently, the use of VR applications in different fields experienced an increased growth over the last years. For the treatment of psychological disorders, various VR therapies emerged since VR creates realistic and interactive environments that are nonetheless safe, easy to adapt and inexpensive. For example, VR has been applied to the treatment of stroke patients [8], different types of mental illnesses such as phobias [9], [10], attention deficit disorder [11], autism [12] and schizophrenia [13], [14]. In addition, there are studies reporting that people experience a feeling of presence when moving within virtual environments [15] and even interact socially with computer-generated characters, called avatars [16]. All those findings together indicate that contemporary computer graphics increasingly succeed in the simulation of virtual characters that appear more and more human-like.

For experimental studies investigating the processing of emotional expressions, virtual faces constitute a major advantage in that they can be easily animated and systematically varied according to the experimenter's needs. There are several VR techniques that have been adopted to create or implement three-dimensional (3D) virtual human face models [17]–[21]. It has been shown in one study that emotions expressed by a virtual face were recognized in a comparable way as emotions expressed by natural facial expressions [18]. Moreover, linear relationships were demonstrated between self-reported valence and arousal and the intensity of virtual facial expressions of anger and happiness [19]. Further, natural and virtual facial expressions elicited comparable activation in the amygdala, a core region in emotion processing. In face-sensitive regions of the brain, however, increased activation was revealed in response to natural faces. The authors interpret this as an indication for the ability of the brain to distinguish between artificial and natural entities [17].

These previous studies investigated different, relatively specific effects of virtual expressions of emotion but only little is known on the differences and similarities of natural and virtual face perception. It would be important to examine specific parameters that might underlie virtual as compared to natural emotion perception and to what extent the synthesized emotional faces address the same psychological and neurobiological mechanisms as natural faces. Additionally, perception of dynamic emotion expressions should be compared between both faces types. As a first step, the current study is dedicated to the validation of a set of virtual facial stimuli in comparison to existing, frequently used facial stimuli of natural emotion. The aim of the study was to create virtual facial expressions that match the well-studied natural facial expressions as closely as possible [22]. Since accuracy in emotion perception is influenced by several factors such as the presentation of the emotional category [23]–[25] or the age of the observer [26], [27], the current study compared recognition rates for six basic emotions separately in subjects of two different age groups (20–39 vs. 40–59 years).

We hypothesized that it is possible to generate virtual emotional expressions that are recognized as well as natural emotional expressions. Regarding the effect of age we expected greater performance decrements with increasing age for virtual than for natural human faces.

## Results

Accuracy rates for the best recognized virtual and natural face for each emotion (happiness, anger, fear, sadness, disgust and neutral) are specified in Table 1. McNemar tests revealed no significant differences between the best virtual and the best natural face for any emotion (McNemar, all *p*>.7) but disgust (McNemar, *p*<.01).

**Table 1:** Accuracy rates in % for best recognized faces.

	Natural face^a^	Virtual face^a^	*p (McNemar)*
Happiness	100	100	>.7
Anger	97.4	87.2	>.7
Fear	84.60	84.6	>.7
Sadness	78.10	84.4	>.7
Disgust	92.3	38.5	**0.004**
Neutral	96.9	100	>.7

a best recognized face.

**Figure 1:**
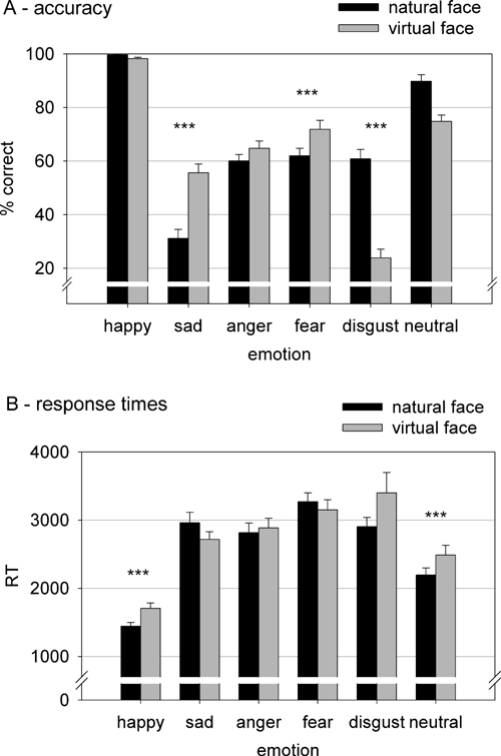
A: Recognition accuracy (chance performance was 16.67%) and B: response times with standard error of mean for natural and virtual facial expressions across all subjects.

After controlling for the effect of the covariate age a generalized linear regression model (probit model) revealed a main effect of *face type* for sadness (z = 3.38, *p*<.001) with an increased relative risk to detect the correct emotion in virtual as compared to natural faces (RR = 2.29). The same factor influenced the recognition of fear (z = 3.06, RR = 1.87, *p* = .002), disgust (z = −4.31, RR = .34, *p*<.0001) as well as neutral expressions (z = −3.81, RR = .78, *p*<.0001). While disgust and neutral expressions were recognized significantly worse in virtual as compared to natural faces, sad and fearful virtual faces achieved significantly better recognition rates than natural faces (see Fig. 1A). As concerns reaction times (RTs), an ANOVA analysis indicated no significant effect of age and gender but a significant interaction between *emotion* and *face type* (F[2.3, 71.2] = 3.96, *p*<.05). Paired t-tests revealed that RTs were significantly slower for neutral (t[31] = 8.38, *p* = .002) and for happy faces (t[31] = 4.03, *p*<.0001) when a virtual compared to a natural face had to be recognized (see Fig. 1B). Furthermore, a correlation analysis discarded the possibility of a speed-accuracy trade-off for any emotion (all *p*>.05); for neutral and happy facial expressions a negative relation between RT and accuracy emerged: the faster subjects responded, the higher was the rate of accuracy (all *p*<.025).

Confusion matrices compared the error pattern of emotion categorization for virtual and natural faces. First, we noted that all faces preferentially communicated the intended emotion except virtual disgust. Second, error distributions for fear (K = .33, p<0.001), sadness (K = .2, p<0.001) and disgust (K = .14, p<0.01) differed significantly between natural and virtual faces. Inspection of the matrices revealed that sadness expressed by human faces was mainly confused with disgust while sadness expressed by virtual faces was mostly mistaken for a neutral face. Human fear was confounded with disgust whereas virtual fear was confused with sadness and neutrality to equal parts. Finally, disgust natural faces were mistaken for anger but virtual faces were mistaken additionally for neutrality (see Table 2).

**Table 2:** Accuracy ratings and confusions (% correct) for virtual and natural faces.

	Ratings					
Expression	Happiness	Sadness	Anger	Fear	Disgust	Neutral
*Virtual faces*						
Happiness	**97.86**	0	0.33	0.82	0.33	0.66
Sadness	0.33	**55.98**	8.14	10.29	4.49	20.76
Anger	2.99	7.65	**65.06**	4.83	9.82	9.65
Fear	5.09	7.95	2.37	**73.94**	3.21	7.45
Disgust	5.61	11.73	31.46	7.65	**22.96**	20.58
Neutral	4.79	11.74	3.97	3.64	0.99	**74.87**
*Natural faces*						
Happiness	**99.67**	0.16	0	0	0	0.164
Sadness	1.00	**31.95**	18.8	8.32	21.63	18.3
Anger	1.18	7.41	**60.44**	9.76	5.39	15.82
Fear	2.69	3.7	8.74	**62.52**	12.44	9.92
Disgust	1.34	12.25	14.43	4.87	**62.25**	4.87
Neutral	2.98	4.97	0.99	0.66	0.17	**90.23**

Boldface indicates recognition rates of intended emotion.

**Figure 2:**
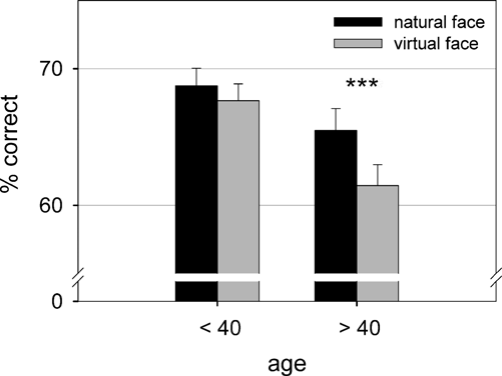
Recognition accuracy with standard error of mean for natural and virtual facial expressions in subjects under and above the age of 40 (chance performance was 16.67%).

As concerns age effects, there was a trend for an interaction between *age* and *face type* (z = −1.79, *p* = .07), which suggested that only people above the age of 40 years had the tendency to recognize virtual facial expressions worse than natural expressions (age >40: z = −3.39, RR = .85, *p*<.001; age <40: z = −.79, RR = .96, *p* = .42). Further, they differed from younger subjects in their mean emotion recognition rate for virtual (z = −3.58, RR = .99, *p*<.001) but not for natural faces (z = −.91, RR = .99, *p* = .36; see Fig. 2). Investigating emotion-specific age effects, there was a main effect of *age group* for virtual expressions of anger (z = −3.23, RR = .98, *p* = .001), fear (z = −2.79, RR = .99, *p* = .005) and marginally for sadness (z = 2.38, RR = 1.01, *p* = .018), even after controlling for computer game experience as a covariate. For anger and fear, subjects above the age of 40 performed significantly worse than younger subjects. For sadness, however, the opposite was found: older subjects outperformed younger subjects in recognition rates.

Computer game experience did not significantly influence the recognition rates of natural or virtual faces; except for the virtual expressions of fear and neutrality, computer game experience was associated with a better recognition rate in males (fear: z = 2.18, *p* = 0.029; neutral: z = 2.72, *p*<0.01), which, however, failed significance after controlling for age. This suggested interaction is plausible since there is a negative correlation between age and computer game experience (r = −3.18, *p*<.05, one-tailed).

## Discussion

The goal of the present study was to investigate whether basic emotions expressed by virtual avatars are recognized in a comparable fashion to emotions expressed by natural human faces. Consistent with previous research, our results indicate that virtual emotional expressions can be generated so that they are recognized as well as natural faces. However, regression analysis revealed a differential pattern among the different emotions. Whereas disgust seemed to be difficult to convey with the current avatar technology, virtual sadness and fear achieved better average recognition rates than natural expressions. Computer game experience did not seem to influence avatar processing directly. However, age effects support the hypothesis that media exposure does influence emotion recognition in virtual faces.

### Specificity to basic emotions

Disgust was the only emotion that could not achieve a satisfying recognition rate in virtual as compared to natural faces. Disgust was mainly confused with the expression of anger. This finding confirms results from earlier studies suggesting that disgust is not recognized consistently within virtual faces [17], [18]. The authors explained this effect with the difficulty to generate AU 9 within virtual faces. AU 9 involves a wrinkling at the base of the nose, which is distinguishing for disgust. Due to low polygon counts at this specific region, it was not possible to consistently achieve wrinkling within the virtual faces [18]. We also experienced this shortcoming during the creation of virtual disgust expressions: AU 9 could not be realistically implemented within virtual faces because the nose region holds only few polygons. With the wrinkling pattern of AU 9 almost missing within the disgust faces, the resulting confusion with the expression of anger is an expected outcome since anger and disgust further share AU 10, 15 and 16. Furthermore, disgust may represent a special case because, as some investigators have argued, it does not belong to the basic emotions but rather represents a mixture of other universal emotions [28]. This mixture of emotions would provide an additional explanation for the difficulty in artificially creating and subsequently detecting disgust within virtual facial expressions. Nevertheless, we suggest that for more complete and naturalistic emotion displays, a higher fidelity of the naso-labial area should be considered for avatar rendering.

Recognition rates were specific for the different emotions. Disgust and neutral faces were more accurately recognized when expressed by a natural face; sad and fearful expressions, in contrast, were rated more accurately when expressed by an avatar. The emotional expressions of anger and happiness were identified equally well in both face types. The recognition advantage for sadness and fear in avatars could be explained by less variability in expressive features, which means by containing less noise. Every natural facial expression consists of frequent and infrequent action units, with the latter also varying among individuals [22]. Specifically, these infrequent action units represent minor emotional cues, which may create noise and lead to less agreement in emotion perception. Since sadness and fear are rather complex emotions with high variability among individuals, it is assumed that those virtual expressions are less noisy and thus pose a recognition advantage over natural faces in the present study. However, when only frequent major cues are present within the face (i.e. within neutral facial expressions) natural faces are better recognized. This ease is also reflected in response times. Response times for natural faces expressing no emotion are faster than for virtual faces.

The pattern of errors that were committed for natural and virtual faces, indicated differences for the same emotions that varied in recognition rates: fear, disgust, and sadness. Notably, in contrast to natural faces, emotions expressed by avatars were predominantly mistaken for neutrality. This feature indicates that emotions expressed by avatars were not confused with completely different emotions as emotions expressed by natural faces. A tendency to rate a face as neutral rather arises when people are unsure about the emotion expressed. This effect is supported by subjective reports of participants indicating a trend towards neutrality when being insecure about the emotion displayed. In future studies subjects may be more clearly instructed to choose neutrality only when they detect no emotional expression within the face.

### Media exposure and age

One could argue that correct emotion perception in virtual faces is facilitated and consequently confounded by computer game experience, i.e. with experience of the outer appearance of avatars. This does not seem to fully explain our data because no direct relationship could be established between the degree of computer game experience and the ability to recognize virtual emotional expressions. Computer game experience was, however, correlated with age. This finding indirectly supports the importance of computer game exposure as one possible factor contributing to older people's decreased performance in the recognition of virtual faces.

When comparing the effects of age on natural and virtual emotion perception it became apparent that the older age group, between 40 and 60 years, showed deterioration only in recognition of virtual facial stimuli. In the present study older participants recognized emotions in natural faces as well as their younger counterparts. This seems to be in contrast with previous research, which suggested an age-related decrease in natural emotion recognition [29]. However, in these studies older participants showing deteriorated emotion recognition were all above the age of 60. One study testing 3 different age groups (younger-aged: ages 21 to 39 years, middle-aged: ages 40 to 59 years, older-aged: ages 60 to 81 years) found the age-related decrease in the ability to recognize emotions only after the age group of 60 [30]. This is in line with our finding that natural stimuli are recognized equally well within the age groups of 20–40 and 40–60 years. The virtual emotions, for which recognition rates decreased with age in our study resemble the same emotions that are known to be identified more poorly by the elderly, namely the negative emotions of fear and anger [26]. This worse performance, however, points less to a decrement in the ability to generally recognize emotions but to unfamiliarity with virtual faces. This unfamiliarity might lead back to the general social and medial learning history of the older generation. Young people today grow up with the computer, the internet and computer games. Hence, they are used to communication in a virtual space, to the appearance of virtual characters online and to virtual interaction [31], [32]. This lack of general experience with a virtual space, more than just experience with computer games, may constitute a factor that makes recognition of emotion more difficult in virtual faces. To further examine this relationship, factors such as experience with computers and internet as well as duration or intensity of computer/internet use should be considered in future studies.

Surprisingly, results also reveal a tendency of the elderly to have superior recognition of sadness, independent of face type. Due to an increasing number of unavoidable losses such as cognitive decline, decline in physical health and death, people of older age may be generally exposed to the emotion of sadness more often than younger people [33]. Furthermore, older people show greater subjective and physiological reactions to sadness-inducing stimuli [34]. We speculate that due to this increased presence of sadness in the lives of older people they are also more sensitive in the recognition of sadness. The PANAS measure of affective state administered in the present study did not reveal an increased subjective feeling of negativity within older people. The negative subscale of this questionnaire, however, is not specifically constructed to assess sadness but includes items like anxiety, nervousness, guilt and hostility. In conclusion, the findings regarding effects of age seem to open promising research possibilities but need replication beforehand because sample sizes for the two age groups were limited.

### Limitations and outlook

Recognition rates of the basic emotions were low in the present study. In particular the negative emotions of anger, sadness, fear and disgust yielded low recognition rates. Previous studies applying facial stimuli from the same stimulus collection showed recognition rates around 80–90% [22], [23], [28], [35]. Conceivably, lower recognition rates are due to the selection of medium intensity emotions as compared to the high intensities, which were used in the previous studies. Indeed, neutral faces, which did not differ in intensity, obtained the same recognition rates around 90% in the present study. Similarly, the most reliably recognized happy facial expressions did not yield lower recognition accuracy at the lower emotion intensity. Recognition accuracy for negative emotions that are easily confused with each other, however, decreased when lower intensities were applied. Nevertheless, the recognition rates of both face types were comparable because intensity ratings were matched for natural and virtual faces.

Our results demonstrate that it is possible to create virtual expressions of the classic basic emotions that are recognized as well as, or sometimes even better than natural emotional expressions. Though, it should be kept in mind that with presenting static facial stimuli the current study only presents a first step in the process of validating the comparability of facial emotional displays in human and virtual faces. As a next step faces should be animated to test the authenticity of dynamic displays of virtual emotion. Furthermore, our results only indicate comparability of the classic basic emotions as described by Ekman [1]. Yet in everyday reality pure basic emotions are encountered only rarely. Facial expressions are rather nuanced by differences in subordinate categories of emotions that are related to more than one basic emotional category [36]. Future research should especially focus on investigating more ambiguous and nuanced emotional expressions. Regarding this aim, virtual reality is very adjuvant because it allows the direct manipulation of facial expressions by systematically changing parameters, combining action units from different emotions and thereby easily creating different nuances of emotions.

Virtual expressions of sadness and fear were better recognized than their natural counterparts. These better recognition rates may be due to the absence of distracting minor emotion cues. Such unambiguous emotional displays can represent an advantage for therapy programs involving patient populations who are impaired in emotion recognition. Virtual faces contain only frequent action units that serve as major cues for the different emotions. This feature makes them a perfect means for learning to distinguish emotions. When patients improve and become able to recognize basic emotional features in the avatars, the level of difficulty can be increased to natural facial emotions that additionally contain various infrequent and non-characteristic action units [22]. Future studies could contribute to the comparison of natural to virtual emotion recognition in patient populations by testing whether virtual faces are consistently better recognized than natural faces.

For clinical applications, the cognitive and neural mechanisms underlying processing of virtual and natural faces should be established because similar recognition rates do not necessarily demonstrate same processing. As concerns the James-Lange model, emotion recognition in faces can be considered in part being conveyed by internal representation of the observed body-state. To confirm this model, physiological reactions such as heart rate, skin conductance and respiration, as well as measures of facial expressiveness to virtual and natural faces could be compared [37]. Furthermore, neural encoding may differ between emotions seen in virtual and natural faces. Only one study has investigated brain activity in reaction to natural and virtual faces [17], and it indicated differential activity. All these factors could be of relevance when studying the aberrant processes underlying impaired emotion recognition in social communication disorders, such as autism or schizophrenia, and help to better understand the underlying deficits.

We conclude that validated virtual emotional expressions will be of major relevance in emotion research and therapeutic settings because animation and change of parameters can be easily performed. The avatars investigated in the present study further have the advantage that they are implemented within a game engine so that they can be easily included into interactive and realistic social environmental scenes. These scenes would provide an excellent tool for investigating the neural processes underlying complex human social behaviour. Moreover, the differential pattern of emotion recognition suggests that some of the processes underlying emotion recognition can be disentangled. Though, before being able to apply virtual faces in a way comparable to natural faces, naso-labial emotion rendering and the balance of major and minor emotion cues still needs improvement.

## Materials and Methods

### Participants

A total of 32 subjects of Caucasian origin took part in the present study. They were recruited through advertisements posted at the University hospital of the RWTH Aachen University. Half of the subjects were between the age of 20 and 40 and the other half between 40 and 60 years of age. The gender distribution was equal in both samples (8 males, 8 females each). All subjects were screened with the German version of the Structured Clinical Interview for DSM-IV, axis I disorders (SCID-I) [38] and were excluded if there was any indication of an existing psychiatric disorder. Accordingly, two subjects of a sample of originally 34 subjects screened positive for cannabis abuse and were excluded from the study. Furthermore, the MWT-B, a German test for verbal crystallized intelligence (Mehrfachwahl Wortschatz Intelligenztest, MWT-B) [39] was administered as well as a questionnaire evaluating computer game experience [40]. Finally, current affective state of subjects was assessed by the Positive and Negative Affect Scale (PANAS) at the beginning of the study [41]. Table 3 shows the relevant demographics of the sample.

**Table 3:** Demographic information on the experimental groups.

	Overall (n = 32)		<40 years (n = 16)		>40 years (n = 16)		*p*
	Mean	±SD	Mean	±SD	Mean	±SD	
Age (years)	38.3	12.4	27.2	5.1	49.4	5.2	-
Education (years)	13.8	3.8	14.5	2.6	13.0	4.6	0.25
IQ (MWT-B)	118.1	11.7	115.4	11.1	120.8	12.0	0.20
PANAS positive	31.1	5.8	29.1	5.2	33.1	5.9	**0.05**
PANAS negative	11.8	4.2	12.6	5.8	11.0	1.0	0.28
Video game experience (%)	28.1	-	43.8	-	12.5	-	**0.05**

MWT-B, Mehrfachwahl Wortschatz Intelligenztest (vocabulary intelligence test); SD, standard deviation; PANAS, Positive and Negative Affect Scale.

The study was approved by the local Ethics commission and performed according to the Declaration of Helsinki. All participants gave written informed consent after having received a full description of the study.

### Facial stimuli

#### Virtual facial stimuli

**Figure 3:**
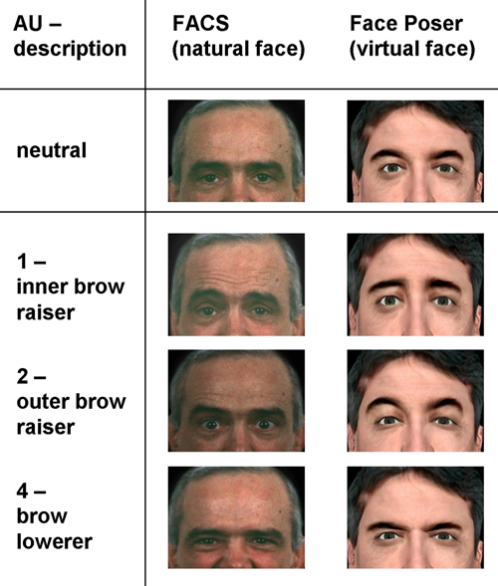
Implementation of action units 1, 2 and 4 in a natural and virtual face.

Virtual facial expressions were created with the Face Poser of the Software Development Kit implemented in the Half-Life 2® computer game (Valve Software, Bellevue, Washington, USA). The implementation of the five basic emotional expressions (happiness, anger, fear, sadness and disgust)-as they were defined by Paul Ekman [1]-as well as neutral emotion was achieved using the description of facial surface changes as explained within the handbook of Facial Action Coding System (FACS) [42]. The Facial Action Coding System is a system developed to taxonomize human facial expression. It describes different action units (AUs), which represent the muscular activity that produces momentary changes in facial appearance. The Software Development Kit used within the present study offers a Face Poser, in which facial expressions can be created activating different muscular action units based on FACS. To create the virtual facial expressions we implemented AUs that were also expressed in natural faces [22]. Table 4 presents an overview of the applied AUs and the corresponding intensities (compare with Tab.1 in [28]) and Figure 3 shows an example for matching AUs in a virtual as compared to natural face is presented.

**Table 4:** FACS of virtual emotions: percentage of faces with respective AU present and mean intensity.

AU	Name	Happiness		Fear		Anger		Sadness		Disgust	
		%	Mean	%	Mean	%	Mean	%	Mean	%	Mean
1	Inner Brow Raiser	100	0.5	100	1.0	0	-	100	0.7	100	0.6
2	Outer Brow Raiser	100	0.5	100	0.6	0	-	0	-	89.5	0.9
4	Brow Lowerer	100	-	0	-	100	1.0	94.7	0.2	0	-
5	Upper Lid Raiser	100	0.5	100	1.0	0	-	0	-	0	-
6	Cheek Raiser	100	0.5	0	-	0	-	0	-	0	-
7	Lid Tightener	100	0.5	0	-	94.7	0.9	36.8	0.1	0	-
9	Nose Wrinkler	100	0.3	0	-	0.3	0.5	0	-	100	1.0
10	Upper Lid Raiser	0	-	0	-	47.4	0.5	0	-	68.4	0.7
12	Lip Corner Puller	100	0.5	0	-	0	-	0	-	0	-
15	Lip Corner Depressor	0	-	100	0.6	94.7	0.9	89.5	0.9	100	0.6
16	Lower Lip Depressor	0	-	0	-	84.2	0.8	0	-	52.6	0.5
17	Chin Raiser	0	-	0	-	36.8	0.2	0	-	0	-
20	Lip Stretcher	0	-	89.5	0.3	0	-	0	-	0	-
23	Lip Tightener	0	-	0	-	0	-	0	-	89.5	0.9
24	Lip Pressor	0	-	0	-	84.2	0.8	0	-	0	
25	Lips Part	0	-	0	-	0	-	21.1	0.1	52.6	0.5
26	Jaw Drop	0	-	100	1.0	0	-	0	-	0	-
38	Nostril Dilator	0	-	0	-	0	-	36.8	0.4	0	-

FACS, Facial Action Coding System; AU, action unit.

**Figure 4:**
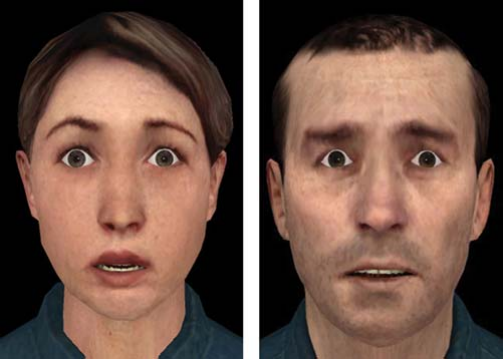
Examples of virtual emotion: fear expression in one male and one female character.

The created virtual facial expressions were validated in a pilot study. For this purpose 42 healthy volunteers recruited in the University Clinic Aachen evaluated the facial material according to the expressed emotion, its intensity level and its naturalness. Intensity level and naturalness were rated on a 6-point scale with 1 representing the impression of “not intense/natural at all” and 6 the impression “extremely intense/natural”. After a validation procedure a final set of 7 female and 12 male avatar characters were chosen for the current study resulting in 114 virtual facial expressions. These facial expressions were all rated as having a medium intensity level with disgust being the emotional expression with the lowest rated intensity and happiness the one with the highest intensity (Table 5). Regarding naturalness all faces were rated moderately natural with mean naturalness scores ranging from 3.3 to 4.35. For an example of a virtual facial expression see Figure 4.

**Table 5:** Mean subjective intensity ratings for virtual and natural faces (on a scale from 1-not intense to 6-very intense).

	Virtual faces			Natural faces		
	*Mean*±*SD*	*Min*	*Max*	*Mean*±*SD*	*Min*	*Max*
Happiness	4.41±0.46	3.41	5.30	4.53±0.86	2.67	5.69
Fear	3.91±0.60	2.43	4.79	3.86±1.04	2.00	5.08
Anger	3.73±0.79	2.38	5.85	3.58±0.94	2.11	5.85
Sadness	3.09±0.57	2.13	4.24	3.35±0.88	2.17	4.50
Disgust	3.46±0.79	1.75	5.16	3.99±0.84	2.17	5.57

SD, standard deviation; Min, Minimun; Max, Maximum.

#### Natural facial stimuli

Photographs of 7 female actors and 12 male actors expressing the five basic emotions and neutral (no emotional expression) were taken from a stimulus set, which has been standardized and used reliably as neurobehavioral probes in emotion research. Development and validation of the facial stimulus material can be found elsewhere [22], [35]. In short, facial stimuli were derived from actors and actresses of diverse ethnicities and ages who were coached to relive appropriate emotion-eliciting experiences of different intensities. In order to match the virtual faces, only natural faces with medium intensity were selected for the current study. For an overview of independently rated intensities of the natural faces see Table 5.

### Experimental task

Facial stimuli were presented to the subjects in 4 different blocks using MATLAB 7.0® (Mathworks Inc., Sherborn, USA). A block consisted of 57 faces of either virtual or natural faces, respectively. The order, in which the blocks were presented to subjects was counterbalanced. Every face was presented for a maximum of 7 seconds or until a response button was pressed. The participants read short instructions indicating that the goal of the experiment was to test how people perceive emotions within facial expressions and that some images would be computer generated while others would be photographs of human faces. They were asked to indicate the emotion depicted by the particular face as spontaneously as possible by choosing one button according to the following categories: happiness, anger, fear, sadness, disgust, or neutral.

### Statistical analysis

Statistical analyses tested whether we can achieve as readily recognizable emotional expressions with virtual as with natural faces. To test for differences between the maximally recognized natural and virtual faces, McNemar tests compared the marginal distributions in the 2×2 table of the recognition rates for each of the six emotions.

To study the influence of different regressors on recognition rates, a generalized linear model (binominal responses in a probit regression model) was computed with *face type* (virtual or natural) and *emotional expression* (happiness, anger, fear, sadness, disgust, or neutral) being the within-subject factors. The *p*-values were Bonferroni-corrected. Effect estimates were given as relative risk (RR) since the GLM approximated proportional risk rates. Moreover, computer game experience was entered into a generalized linear model for human and avatar faces separately. Participant's gender and age were considered as covariates. Response times can be expected to be asymptotically normal distributed and, therefore was analyzed in a linear model (repeated measure analysis of variance; paired t-test) applying the same independent variables.

Finally, confusion matrices for the error responses were calculated and distributions were compared using the Kolmogorov-Smirnov test for two samples. Statistical analyses were performed with SPSS® (SPSS inc., Chicago, USA) and MATLAB 7.0® (Mathworks Inc., Sherborn, USA).
