# Correction: Recognition Profile of Emotions in Natural and Virtual Faces

**DOI:** 10.1371/annotation/b1a62b84-2d44-4250-b985-915211361ce2

**Published:** 2008-11-22

**Authors:** Miriam Dyck, Maren Winbeck, Susanne Leiberg, Yuhan Chen, Ruben C. Gur, Klaus Mathiak

There was an error in the fifth author's name and affiliation. The fifth author's name should be Ruben C. Gur and he is additionally affiliated with Philadelphia Veterans Administration Medical Center, Philadelphia, Pennsylvania, United States of America. 

